# Supplementary material for: Are carbon emissions trading and green financial instruments synergistic? -Comprehensive quantitative research based on content analysis
Source: PLoS One. 2024 Mar 7;19(3):e0298601. doi: 10.1371/journal.pone.0298601 (PMC10919601; doi:10.1371/journal.pone.0298601)
Supplement: S1 Data — (ZIP) [file pone.0298601.s001.zip › Data/地级市Z_F工作报告.zip!/╔╜╢1⁄2/▒⌡╓▌╩╨/└┤╫╘╣1⁄2╓┌║┼í╛┬φ┐╦╩2╛▌═°í┐_2010.docx]

一、2009年工作回顾

2009年，是新世纪以来我市经济社会发展最为困难、形势最为严峻的一年；是全市上下坚决贯彻党中央、国务院保增长、保民生、保稳定的一系列政策措施，万众一心、共克时艰的一年；是全市人民以胡锦涛总书记、温家宝总理视察滨州为强大动力，在省委、省政府和市委的正确领导下，迎难而上、硕果累累的一年，较好地完成了市九届人大三次会议确定的目标任务。

——积极应对金融危机，经济保持平稳较快增长。预计全市实现生产总值1346亿元，增长13%以上。三次产业比例调整为10.1：59.3：30.6.。规模以上工业完成增加值763.5亿元，增长15.6%。规模以上固定资产投资完成723.4亿元，增长24.7%，城镇居民人均可支配收入17500元，增长9.6%；预计农村居民人均纯收入6200元以上，增长10%左右。完成地方财政收入80.17亿元，增长13.9%。实现社会消费品零售总额360.3亿元，增长19.1。金融机构各项人民币存、贷款越分别达到918.3亿元和847亿元，增长31.8%和30.3%。全市共争取中央扩大内需新增资金4.2亿元，争取省调控资金17亿元。

——切实转变发展模式，经济发展质量效益不断提高。预计规模以上工业企业实现利润150.9亿元、利税252.8亿元，分别增长19.6%和20.3%。规模以上企业达到1559家，主营业务收入过百亿元的企业达到6家。完成工业技改投入248.7亿元，增长83.6%。新认定高新技术企业8家，高新技术产业产值占规模以上工业总产值的比重达到16.3%。新增中国驰名商标2件。1家企业获得首届省长质量奖。预计民营经济实现增加值850亿元。新增商业银行1家、小额贷款公司3家。全年实现旅游总收入33.3亿元，增长27%。预计服务业实现增加值414.3亿元，完成投资增长53.6%，跻身全省服务业发展先进市行列。预计万元地区生产总值能耗降低5.83%，二氧化硫和化学需氧量减排完成“十一五”总任务的90%。

——大力推进“粮丰林茂、北国江南”建设，农业农村经济健康发展。农业牧业渔业总产值达到262.9亿元，增长4.5%。粮食总产295万吨，增长6.6%，连续七年实现增产。林水会战取得重大突破，完成各类水利工程430处，新增蓄水能力3500万方；新增合格造林面积14.3万亩；完成市级标准化方田9万亩。黄河标准化提放第三阶段工程全面竣工。建成现代化农业示范园29家，新增农业标准化基地5万亩，农产品质量明显提升。规模以上农业龙头企业达到476家，新发展农业专业合作社340家。全面落实强农惠农政策，发放粮食直补和良种、农机等涉农补贴5.3亿元。培训农民10万人次。农村信息化平台正式启动。364个新农村示范场建设达标。圆满实现省政府下达的耕地保护与美国白蛾防控目标。

——着力实施“基础设施建设年”活动，支撑能力明显提升。滨德高速滨州段路基和中小桥涵工程基本完工。疏港公路一期工程全部路基、桥涵及东半幅路面工程，二期工程顺利推进。滨州客运总站落成启用。孙武黄河大桥获得国家发改委立项核准。滨港公路一期工程正式运营，二期工程通过省发改委审批。德龙烟铁路开工建设。滨州港2个3万吨级散杂货码头及配套工程开工建设，11公里引堤工程基本完工。护砌加固防潮提10公里。黄骅至滨州500千伏电网工程全面完成。城市规划建设管理取得新突破，市区建设完成投资9.5亿元，新增绿化面积193平方米，建成城市道路60公里，铺设雨污管线101公里。完成旧城改造、城中村改造拆迁面积59万平方米，启动拆建整村140个，新建农村住房2.8万户，改造危房5443户。大力开展城乡环境综合整治活动，城市精细化管理水平明显提高。

——不断深化改革开放，发展活力进一步增强。行政管理体制改革、事业单位人事制度改革和国有企业改革有序推进。市县政府机构改革、文化体制改革、医药卫生体制改革正式启动。市级财政国库集中支付改革全面推进。惠民县、阳信县被纳入“省直管县”财政体制改革试点。对外贸易趋向好转，全年实现进出口总额33.7亿美元；实际到账外资1.7亿美元。第八届银企合作促进会签约资金945.9亿元。中国玉米油股份有限公司在香港挂牌上市，融资7.2亿港币。滨化集团、齐星铁塔、鲁丰铝箔通过中国证监会上市审核。赴北京、香港等各类招商活动成效明显。县域经济快速发展，帮扶阳信、惠民成效显著。滨州经济开发区被核准为全省科学发展园区，高新技术产业开发区调整设立，北海新区筹建工作扎实推进。

——高度关注民生，群众生产生活条件持续改善。新增城镇就业4.2万人，新增农村劳动力转移就业8万人。城镇登记失业率3.14%。城市低保标准人均每月提高74元，农村低保标准人均每年提高318元，惠及城乡低保对象10.6万人。敬老院建设任务全面完成，农村五保户集中供养率达到85.9%。城镇居民基本医疗保险参保人数达到25万人，新型农村社会养老保险试点开始启动。开工建设经济适用房26万平方米、廉租房8.8万平方米。解决和改善了农村33万人的饮水安全问题。建设农村公路1207公里，维修改造闸带桥57座，新建改建农村公路桥梁92座。完成48处学校危改项目，农民中小学“两热一暖一改”试点工程全面竣工。救助贫困学生4.6万名。城乡社区卫生服务实现全覆盖，新农合参合率达到98.8%。帮助650名残疾人实现就业，帮扶1000户贫困残疾人家庭实现脱贫，为1.4万名残疾人提供了康复服务。

——繁荣发展社会事业、和谐稳定局面得到巩固。举全市之力，成功承办了第十一届全运会武术套路、柔道两项赛事，奥林匹克体育馆被评为优秀场馆。素质教育深入实施，高等教育、职业教育迈出新的步伐。科技创新能力不断提高，被评为全国科技进步先进市。市文化中心开工建设。大型现代吕剧《杨广和》荣获全国“五个一工程”优秀作品奖。成功举办了首届海峡两岸孙子文化交流研讨会、第七届国际家纺文化节等重大节会。市老干部活动中心及老年大学完成主体工程，渤海革命老区纪念园建成开放，市档案馆落成使用。甲型H1N1流感和手足口病得到有效控制。各项人口责任指标圆满完成。红十字工作进入全国先进行列。双拥共建巩固发展，国防动员、人民武装、人民防空工作不断加强。安全生产形势总体稳定，食品药品监管能力进一步提高。对口援建禹里乡的七项工程全部竣工。妇女儿童、广播电视、新闻出版、民族宗教、外侨、物价、仲裁、对台、史志、慈善、地名、地震、气象、海事、水文、油区管理等各项工作都取得新成绩。

——认真开展“发展环境优化年”活动，政府行政能力切实提高。深入学习实践科学发展观，政府职能进一步转变。认真办理人大代表建议67件、政协委员提案252件，加强与各民主党派、工商联、无党派人士和人民团体的联系。政府信息化、行政审批电子监察系统开始运行。取消、下放行政许可事项35项，受理市长公开电话4.9万件、市长信箱来信940件，解决了一批群众关心的热点难点问题。各级各类应急队伍相继建立，应急管理体制日趋完善。强化监察和审计监督，110人次受到行政问责，反腐倡廉建设取得新成效。

各位代表，回首惊心动魄的2009年，面对国际金融危机的严重冲击，全市人民和衷共济，沉着应对，攻坚克难，圆满完成新中国建立60周年和十一运安保艰巨任务，滨州赛区被十一运组委会评为全省四个最佳赛区之一，我市运动员实得金牌数列全省第三位，邹平县连续六年进入全国县域经济基本竞争力百强县，博兴县首次跨入百强县行列，魏桥创业集团销售收入突破800亿大关，中国优秀旅游城市、省级园林城市创建成功，各条战线捷报频传，各项工作再创佳绩。实践再次证明，任何困难都动摇不了我们加快发展的决心，阻挡不了滨州人民追赶超越的步伐。只要我们团结一心，锐意拼搏，不为任何风险所惧，不为任何干扰所惑，就一定能够战胜前进道路上的各种艰难险阻，就一定能够夺取一个又一个的新的更大的胜利！

成绩来之不易，令人鼓舞。这得益于省委、省政府和市委的正确领导，得益于市人大、市政协及社会各界的监督和支持，得益于全市人民的科学实干、共同努力。在此，我代表市人民政府，向辛勤工作在各条战线的广大干部群众，向离退休老同志，向各民主党派、工商联、各人民团体和社会各界人士，向驻滨人民解放军、武警官兵和中央、省驻滨单位，向所有关心、支持、参与滨州改革发展的投资者、海内外朋友，表示衷心的感谢并致以崇高的敬意。

在肯定成绩的同时，我们也清醒地认识到，全市经济社会发展和政府工作中还存在一些突出的矛盾和问题。一是规模以上工业企业户数少，民营经济活力不足，中小企业发展缓慢。二是传统农业比重大，现代农业亮点少、规模小，产业化程度不高，农业增收后劲不足。三是结构性矛盾依然突出，三次产业比例和不尽合理，现代服务业发展相对滞后，财政收入对重点工业企业依赖性较大，城镇化水平较低，区域经济发展还不够平衡。四是重大基础设施建设和相对薄弱，港口、铁路、公路、水利等规模较小，整体功能较低。五是许多民生问题还没有得到根本解决，影响社会稳定的隐患还没有完全化解，发展环境有待进一步优化。六是个别政府部分的工作还不能适应新形势新任务的要求，过分强调部门利益的现象仍不同程度的存在，少数干部工作标准不高、责任心不强、工作效率偏低。

这些矛盾和问题，有些是改革发展到一定阶段必然出现的，有些是改革不到位衍生的，有些是缺乏工作经验、操作不规范造成的；有些是金融危机冲击下暴露出来的老问题，也有些是在加快发展中产生的新矛盾。无论什么样的矛盾和问题，我们都应该实事求是地去面对，客观公正地去分析，开诚布公地去讨论，真心实意地去解决。既不能因害怕矛盾和问题而不去发展，也不能因强调发展而回避矛盾和问题，真正把解决矛盾和问题的过程，变为了解民意、凝聚民心的过程，变为转变作风、改进工作的过程。只有这样，我们才能保持清醒、趋利避害，才能牢记使命、不负重托，才能集中精力、奋勇赶超，不断开创全市经济社会发展新局面。

二、2010年政府工作的目标和任务

今年是全面完成“十一五”规划的最后一年，改革发展稳定的任务艰巨繁重，做好今年的工作意义重大。今年是我市抢抓机遇、追赶超于的关键一年。黄河三角洲高效生态经济区开发建设正式上升为国家战略，山东半岛蓝色经济区规划建设顺利推进。两大经济区相互叠加，相互融合，为我市科学发展、加快发展搭建了良好平台，提供了千载难逢的历史机遇。

也是滨州撤地建市十周年。十年磨一剑，十年大跨越。建市十周年的历史，是一部励精图治、锐意进取的奋斗史，是一部矢志不渝、顽强拼搏的创业史，是一部开拓创新、谱写辉煌的发展史。十年前，面对历史机遇，滨州人民拉开了加快发展的序幕；十年后的今天，时代需要我们继续吹响“追赶超越”的号角。站在新的历史起点上，只要我们以更大的勇气和毅力，乘势而上，奋斗向前，我们的事业一定会兴旺发达，滨州的明天一定会更加美好！

政府工作的总体思路是：以科学发展观为统领，全面贯彻党的十七大、十七届三中、四中全会和中央、省、市经济工作会议精神，抢抓黄河三角洲高效生态经济区开发、山东半岛蓝色经济区建设的重大历史机遇，继续深化“基础设施建设年”、“发展环境优化年”，大力开展“项目建设年”、“质量效益年”活动，着力抓投入促增长，抓调整促转型，转统筹促协调，抓改革促开放，抓基础促配套，转民生促和谐，努力完成“十一五”规划的目标任务，进一步推动全市经济社会又好又快发展。

今年全市经济社会发展的主要目标是：生产总值增长13%，规模以上固定资产投资增长23%，地方财政收入增长12%，规模以上工业增加值增长14.5%，社会消费品零售总额增长17.5%，外贸进出口总值增长12%，城镇居民人均可支配收入、农村居民人均纯收入均增长10%，居民消费价格涨幅控制在3%左右，城镇登记失业率控制在3.6%以内，人口自然增长率控制在5.5‰以内，万元地区生产总值能耗下降3.8%，二氧化硫排放减少6.1%，化学需氧量排放降低2.15%。

以上的目标任务，做好今年的政府工作，必须牢牢把握以下几点。一是坚定不移地坚持解放思想。能不能抓住机遇、加快发展，关键取决于思想解放的力度和程度。把解放思想作为各项工作的先导，真正体现在工作上，落实到行动中，在应对挑战中敢于突破常规，在抢抓机遇中勇于超越自我，以思想大解放推动经济社会大发展。二是坚定不移地坚持改革创新。把勇争一流作为追赶超越的根本要求，树立强烈的竞争意识，增强改革创新的勇气、坚韧不拔的斗志、昂扬奋发的干劲，努力使各项工作提高到一个新的水平。三是坚定不移地坚持调整结构。把转方向、调结构作为保持经济平稳较快发展的根本举措，在发展中促转变，在转变中谋发展，不断推动产业结构优化升级，努力形成以高效生态农业为基础、环境友好型工业为重点、现代服务业为支撑的产业体系。四是坚定不移地加大投入。把项目建设作为增投入、抓发展、促超越的强力支撑，千方百计对上争取项目，全力以赴对外引进项目，集中智慧对内激活项目，以项目推动产业升级，以项目提升综合实力，以项目促进追赶超越。五是坚定不移地坚持统筹协调。把建设和谐社会作为重要责任，着力促进城乡、区域、经济社会、人与自然协调发展，突出以人为本，切实把财力、物力、精力向社会事业、民生保障倾斜，让人民共享改革发展的成果。

三、2010年政府主要工作

围绕政府工作的主要目标和总体思路，今年突出做好十个方面的重点工作。

（一）加快推进黄河三角洲高效生态经济区和山东半岛蓝色经济区开发建设。纵观中外发展，大江大河三角洲的成功开发，无不有力地带动了所在区域的经济发展。黄河三角洲作为我国最后一个待开发的大河三角洲，后发优势明显，发展潜力巨大。加快黄河三角洲高效生态经济开发区建设，是落实科学发展观、建设生态文明的重大战略决策，是区域发展道路、发展模式的新探索，必将对山东乃至全国科学发展起到积极的示范带动作用。

作为黄河三角洲高效生态经济区面积最大、人口最多的行政区，具有良好的开发基础。土地资源优势突出，拥有未利用土地254.5万亩，具有吸纳要素集聚的独特优势。地理位置条件优越，位于京津冀都市圈和山东半岛的结合部，是环渤海地区的重要组成部分、济南省会城市群最近的出海通道，具有深化区域合作的有利条件。自然资源较为丰富，石油、天然气地质储量约6亿吨和164亿立方米，地下卤水静态储量约460万吨，风能、地热、海洋等资源丰富，具有转为经济优势的巨大潜力。产业发展基础较好，具有一批竞争力较强的支柱产业、实力较为雄厚的骨干企业和市场占有率较高的知名品牌，特色产业初具规模。生态系统独具特色，具有大规模发展生态种植业和养殖业、培育生态产业链、发展生态旅游的优越条件。充分发挥好这些优势，我们完全有条件成为黄河三角洲高效生态经济区开发建设的主战场。

高效生态经济区发展规划要求，结合编制“十二五”规划，加快编制完成实施意见和各项专项规划，科学确定区域工作定位和产业发展布局。强化基础设施支撑，疏通区域内外陆海通道，加快较大交通设施建设，完善立体交通体系。以沿海为开发重点，以园区为发展载体，以内陆腹地为发展空间，做好与山东半岛蓝色经济区规划建设的有机结合，找准突破口和切入点，精心组织一系列宣传推介和招商引资活动，谋划一批列入国家规划的重大项目。创新体制机制，力争在财税、投融资、土地、海域等领域率先出台一批先行先试的政策和制度。加强组织领导，明确分工，落实责任，优化环境，力争用10年时间，打造高效生态经济示范区、特色产业集群和循环经济示范基地，努力使滨州成为环渤海地区隆起的又一重要区域。

（二）加快推进工业调整振兴。工业是转方式、调结构的重点所在，必须深入实施“工业兴市”战略，不断提高工业的综合竞争力。

推动产业集群发展。高度重视传统产业升级改造，不断提升纺织、化工、机械制造、粮油加工等产业的技术含量和产品附加值，开发时尚、健康、个性化高档家纺服装产品，培育自主品牌，促进产业链条延伸配套，推动骨干企业由大变强。重点扶持新能源、新材料、新医药、新信息、海洋工程等高新技术产业，加快培育一批成长性高、带动力强的骨干企业，开发一批具有自主知识产权的核心产品，建设以新型油化工、盐化工为特色的新材料产业基地。扶持壮大中小企业，加快培育区域特色产业集群。

促进工业经济提质增效。坚持走新型工业化道路，不断推动信息化与工业化的融合。大力发展循环经济，探索发展低碳经济，全面推行清洁生产，严格落实节能减排责任制，一手抓淘汰落后产能，一手抓源头污染控制，扎实推进国家环保模范城市创建和生态城市建设，确保完成“十一五”节能减排目标。

加强企业家队伍建设。企业家是现代企业的灵魂，对推动经济发展和社会进步负有重大责任。营造企业家健康成长的良好环境，努力培养一批具有战略眼光、善于把握未来、敢于开拓创新的优秀企业家群体。积极引导广大企业家带头干事创业，勇于承担社会责任，投身社会公益事业，维护职工合法权益。

（三）加快推进“三农”工作。农业稳则全局稳。按照“粮丰林茂，北国江南”的要求，加大统筹城乡发展力度，夯实农业农村发展基础，积极打造黄河三角洲高效生态农业示范区。

把加快农业现代化发展步伐作为农业工作的重中之重加强基础设施建设，调整优化农业结构，改变传统农业耕种模式，深入开展粮食高产创建活动。大力发展高效生态农业，积极建设生态畜牧业和生态渔业基地，提高农业专业化、集约化水平。深化农业产业化经营，发展壮大农业龙头企业和农民专业合作组织。高度重视动植物疫病防控工作，保障农产品质量安全。

把林水会战作为改善农民生产生活条件的关键措施，大力实施绿色通道精品工程。积极发展耐碱抗旱、优质高产、生态安全种植业，建设优质粮食、棉花、特色林果、蔬菜生产加工出口基地，完善产业链条，提高标准化水平。按照省政府要求，完成“ 南水北调”工程前期准备和建设任务。

把新农村建设作为农村经济平稳较快发展的持久动力，全面落实各项强农惠农政策，切实减轻农民负担，维护农民合法权益。不断拓宽渠道，促进农民增收。加强农业科技服务，加快农村信息化进程。推进“万村千乡”市场工程，扩大农村消费。规范农村土地经营权流转，稳妥推进林权制度改革，加快小型水利工程产权制度改革，大力整治农村环境，搞好垃圾污水处理，建设新农村示范村410个。

（四）加快推进城镇化进程。城镇化是扩大内需、推动经济增长的强劲动力，是优化经济、社会和城乡结构的必由之路。

充分发挥规划的先导作用，按照统筹规划、合理布局、完善功能的原则，积极推进新型城镇化建设，以小城镇和中心村建设为重点，提升县城和中心村的承载辐射能力，积极稳妥开展迁村并居，加快推进城乡一体化进程。放宽政策，降低门槛，促进特色产业、优势项目向县城和重点镇集中，吸纳农村人口向小城镇集中，引导居民向社区集中。把加快县域经济发展放在更加突出的位置，着力增强发展内生动力，全面提高综合实力。切实提高帮扶阳信、惠民工作的实效，努力缩小南北差距，促进全市经济社会协调发展。

加强中心城市建设管理，巩固完善新城区，改造提升老城区，着力实施一批城市道路、配套设施、特色城市街区重点工程。继续做好道路、水系、街头绿地、公园广场等绿化建设，升级改造中海、蒲湖风景区，积极争创国家级园林城市。加快城市管理信息化建设，严格落实网格化管理，进一步完善城管执法长效机制。强化对房地产市场的监管，推动房地产业平稳健康发展。

（五）加快推进重大基础设施建设。把基础设施建设作为落实国家、省重点区域发展带动战略的强力保障，突出抓好公路、铁路、港口等重大工程建设，为经济社会发展提供重要支撑。

加快交通公路建设。完成滨德高速公路滨州段路基、桥涵及部分路面工程。疏港公路确保一期工程完工，二期工程实现简易通车。开工建设一批国道、省道路网改建项目。继续做好环渤海高等级公路、长深高速滨州至东营段前期工作。开工建设孙武黄河大桥。

加快铁路建设。积极配合做好山东“第五纵向铁路运输通道”项目的论证、规划等前期工作，争取将滨港铁路二期工程列入国家发展计划。完成德龙烟铁路滨州段征地和拆迁，开工建设路基、桥涵等工程。适时开展黄大铁路征地和拆迁工作。

加快港口建设，进一步完善港口基础功能，推进滨州港2个3万吨级码头主体建设。力争完成防波堤和临港防潮堤一期主体工程。积极推进中聚能源9个5000吨级码头及大型物流园区项目建设。

加快北海新区筹建步伐。尽快编制完成北海新区概念性规划以及水电路讯等专项规划，强力推进起步区基础设施建设，努力建成全市科学发展的实验区。

（六）加快推进服务业繁荣发展。服务业是产业结构优化升级的主要方向。继续抓好重点城区、重点园区、重点企业“三大载体”建设，大力发展生产性服务业，积极发展消费性服务业，促进服务业拓宽领域、扩大总量、增强后劲、提升层次，构筑特色鲜明的现代服务业体系。

以打造黄河三角洲金融服务中心为目标，积极争取设立地方性银行，吸引更多的国内外金融机构在滨州设立分支机构。加快发展证券、保险、信托、租赁等现代金融服务，支持担保机构健康成长。

以打造鲁北物流中心为目标，依托得天独厚的区位优势，发挥国家级公路运输主枢纽的作用，加快二三产业分离，积极建设东城商贸物流、开发区环渤海现代物流、邹平工业物流和北海新区临港物流等“四大物流园区”，培育壮大京博物流、西王物流、滨阳工贸等一批骨干企业。提高物流信息化合标准化水平，大力发展第三方物流。

整合旅游资源，提升旅游产业竞争力。丰富拓展“孙子故里”、“生态滨州”文化旅游品牌体系，重点发展以“文化采摘”为载体的休闲观光旅游，尽快实施一批民俗旅游、红色旅游、工业旅游、生态旅游等重点旅游项目，推介一批精品线路，促进旅游产业做大做强，扩大滨州旅游的知名度和影响力。

（七）加快推进改革开放。深化重点领域、关键环节的改革，拓展对外开放的广度和深度，为推动科学发展释放新能量，为实现追赶超越增添新动力。

认真落实“三定”方案，完成市县政府机构改革任务，深化医药卫生体制改革，完成各项刚性指标。稳步推进财税体制改革，创新投融资管理体制机制，规范各级各类投融资平台，不断增强政府投融资能力。推进文化体制改革，加快经营性文化单位转企改革，全民实施公益性文化单位内部机制改革。

优化进出口商品、市场和贸易方式结构，组织企业参加境内外重点展会，扩大国际市场占有率，推动加工贸易转型升级。大力实施“走出去”战略，抓好欧亚木器在俄罗斯的森林开放增资项目、京博集团在新加坡并购等重点项目。积极推动企业上市，进一步扩大融资规模。加快高新技术产业开发区和8个省级经济开发区发展步伐。

强力推进招商引资。项目是招商之本，不断增强项目对外商的吸引力，提高招商引资的成功率。突出招商引资的针对性和实效性，创新招商方式，落实优惠政策，加大赴日韩和港台招商力度，做好对接央企的文章，进一步落实重大项目联席会议制度，加大对签约项目的跟踪服务力度，提高项目履约率和资金到位率。

（八）加快推进各项社会事业发展。坚持经济社会协调发展，立足于确保公平、促进均衡，不断加大投入力度，更好地满足群众基本公共服务需求。

繁荣发展文化体育事业，充分发挥滨州文化资源优势，发展一批以数字、网络等高新技术为支撑的新兴文化业态，培育壮大一批以影视传媒、新闻出版、文艺演出、文化创意为主体的支柱产业。加大文明创建力度，提高公民文明素质。加强文化市场综合执法管理，深入开展“扫黄打非”行动。实施全民健身计划，广泛开诊群众文体活动。全力备战省二十二届运动会，力争竞技体育水平继续保持全省前列。

深入实施“科教兴滨”战略，重点建设一批国家、省级实验室和企业技术中心，促进科技成果向现实生产力转化，带动相关领域技术水平整体提升。全面推行素质教育，加快中小学标准化建设。完善义务教育经费保障机制，落实“以县为主”管理体制。发展特色职业教育，提升高等教育办学质量，加强人力资源建设，建立区域人才发展专项资金，培养引进发展高效生态经济所需的国内外优秀人才。

健全突发卫生事件预警防控体系努力控制各种传染疾病暴发流行。加强新型农村合作医疗和城市社区卫生服务规范化管理，新农合参合率达到99%以上。认真组织开展第六次人口普查。全面完成出生人口控制计划指标。

（九）加快推进社会和谐稳定。维护社会和谐稳定是政府工作的重要责任，务必时刻保持清醒头脑，广泛开展平安创建活动，努力保障人民群众生命、财产安全，为经济社会平稳较快发展营造和谐稳定的良好环境。

强化社会治安综合治理，深入推进社会矛盾化解、社会管理创新、公正廉洁执法三项重点工作，切实维护社会稳定。建立推行信访风险评估制度。落实安全监管措施，有效防范和坚决遏制重特大安全事故。加强食品药品市场监管，维护群众饮食用药安全。加快应急指挥平台建设，提高预防和处置各类突发事件的能力。高度重视油区工作，加强油地共建。深入开展普法教育，加强军政军民团结，加快城乡社区建设步伐，全面完成对口元件的各项任务，努力做好民族宗教、妇女儿童、对台、外侨、档案、人防、防震、水文、气象、史志、红十字、慈善等工作。

（十）加快推进民生改善。民生连着民心，民心凝聚民力。我们一定要以对人民群众高度负责的精神，把保民生紧紧抓在手上，竭尽全力做好“十大民心工程”。

1.业促进工程。新增城镇就业3.5万人，新增农村劳动力转移就业5万人。创建3个创业就业园区，新增20家大学生就业见习基地。

2.困难群体救助工程。资助家庭困难学生4.7万人。为享受低保的重度残疾人每人每月发放50员生活补贴。社会福利中心、市儿童福利院建成启用。

3.保障惠民工程，完成城镇居民基本医疗保险制度，积极推进新型农村社会养老保险试点，符合条件的城乡困难群众实现应保尽保。农村低保标准每人每年不低于1200元。

4.饮水安全工程。解决和改善24万农村居民的饮水安全问题。

5.保障工程。全市新增廉租房7.5万平方米，开工建设经济适用房20万平方米。

6.路桥改造工程。新建改建农村公路460公里，改造农村危桥62座，维修改造闸带桥37座。

7.设施建设工程。力争市文化中心完成主体建设，奥林匹克公园全民健身馆、综合训练馆及市体校全面竣工，完成乡镇综合文化站、农村文化大院建设任务。

8.卫生保障工程。新农合人均补助标准不低于100元，城乡居民健康档案建档率达到90%。加快市人民医院、中心医院业务楼建设，市公共卫生中心完成主体工程。

9.水蓝天”工程。加强环境监督，确保全市饮用水源地水质达标率到100%。辖区河流断面水质达到省空目标要求，空气质量不断改善。

10产品质量提升工程。农业标准化基地达到165万亩以上，无公害农产品、绿色和有机食品认证达到260个。

承诺贵在践行，实事务求办好。尽管万亩经受了金融危机的严重冲击，财力还很艰难，但是，对民生的投入只能增加不能减少，保障民生的覆盖面只能扩大不能缩小，群众生活水平只能提高不能降低，努力谱写滨州人民美好生活的新篇章！

四、切实加强政府自身建设

坚持把政府自身建设放在更加突出的位置，不断增强依法、科学、民主行政的能力，更好地为发展、服务、为基层服务、为群众服务。

（一）着力提高行政效能。以市县政府机构改革为契机，深化行政审批制度改革，规范行政权力，减少办事层次和环节，坚决杜绝一件事情让群众炮烙跑去、领导批来批去、部门转来转去的推诿扯皮现象。凡是有利于发展、有利于人民的事情，说了算、定了干，敢抓敢管，切实提高政府工作效率。

（二）全面推进依法行政。加强民主法制建设，严格按照法定权限和程序行使权力、履行职责。政府工作人员无论职务高低，都要自觉接受人大的法律监督和政协的民主监督，做依法行政的模范。严格落实行政执法责任制和过错责任追究制，积极推进市、县（区）行政审批电子监察系统建设，进一步推进行政问责的制度化、规范化。

（三）不断推动管理创新，应用现代信息和通信技术，加快政府信息化建设，推动政府职能转变和管理创新，拓宽政府与公众的沟通渠道，让广大群众有机会讲话、有地方说理，增进公众对政府工作的了解和监督，提高政府工作的透明度，推进政府决策的民主性，科学性和规范性。

（四）大力转变工作作风。执行力是检验工作作风的重要标准。进一步增强加快追赶超越的忧患意识、责任意识，让有令必行成为政府人员的自觉行动，实际工作中敢于直面问题，用好的工作作风促进各项工作的落实，决不让任何一个机遇在我们面前丧失，决不让任何一个项目在我们手中延误，决不让任何一位投资者在我们这里受到冷落！

（五）切实加强廉政建设。严格落实党风廉政建设责任制，让廉政勤政成为政府工作人员的习惯，每个政府工作人员都应做到一要干净，二要干事。要从源头上预防和治理腐败，强化对财政资金和重大投资项目的监察审计，对各类违法违纪行为严肃查处，进一步树立政府为民、务实、清廉的良好形象。

各位代表！雄关漫道真如铁，而今迈步从头越。让我们更加紧密地团结在以胡锦涛同志为总书记的党中央周围，在市委的坚强领导下，进一步解放思想，抢抓机遇，开拓进取，科学实干，奋力书写黄河三角洲高效生态经济区开发建设的壮丽诗篇，为建设富裕、文明、和谐的新滨州而努力奋斗！
